# FHF1 is a bona fide fibroblast growth factor that activates cellular signaling in FGFR-dependent manner

**DOI:** 10.1186/s12964-020-00573-2

**Published:** 2020-05-01

**Authors:** Martyna Sochacka, Lukasz Opalinski, Jakub Szymczyk, Marta B. Zimoch, Aleksandra Czyrek, Daniel Krowarsch, Jacek Otlewski, Malgorzata Zakrzewska

**Affiliations:** 1 Department of Protein Engineering, Faculty of Biotechnology, University of Wroclaw, Joliot-Curie 14a, 50-383 Wroclaw, Poland; 2 Department of Protein Biotechnology, Faculty of Biotechnology, University of Wroclaw, Joliot-Curie 14a, 50-383 Wroclaw, Poland

**Keywords:** FGF, FHF, FGFR, Signal transduction, Cell proliferation, Apoptosis, Glucose uptake

## Abstract

**Abstract:**

Fibroblast growth factors (FGFs) via their receptors (FGFRs) transduce signals from the extracellular space to the cell interior, modulating pivotal cellular processes such as cell proliferation, motility, metabolism and death. FGF superfamily includes a group of fibroblast growth factor homologous factors (FHFs), proteins whose function is still largely unknown. Since FHFs lack the signal sequence for secretion and are unable to induce FGFR-dependent cell proliferation, these proteins were considered as intracellular proteins that are not involved in signal transduction via FGFRs. Here we demonstrate for the first time that FHF1 directly interacts with all four major FGFRs. FHF1 binding causes efficient FGFR activation and initiation of receptor-dependent signaling cascades. However, the biological effect of FHF1 differs from the one elicited by canonical FGFs, as extracellular FHF1 protects cells from apoptosis, but is unable to stimulate cell division. Our data define FHF1 as a FGFR ligand, emphasizing much greater similarity between FHFs and canonical FGFs than previously indicated.

Video Abstract. (MP4 38460 kb)

**Graphical abstract:**

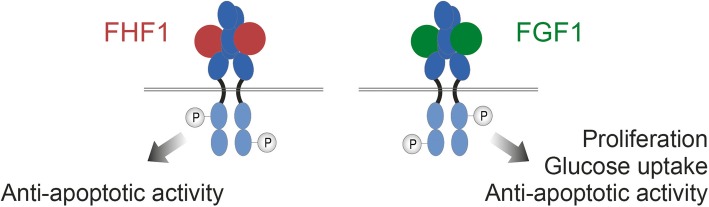

## Background

The fibroblast growth factor (FGF) superfamily consists of 22 genes in humans encoding structurally related polypeptides [1]. Mammalian FGFs are divided into three groups: the canonical FGFs (including FGF1–10, FGF16–18, FGF20, FGF22), the endocrine FGFs referred as FGF19 subfamily (FGF19, FGF21, FGF23) and the intracellular FGFs (FGF11–14) known as FGF homologous factors (FHFs) [1]. First two groups are secreted proteins that act through tyrosine kinase cell surface receptors (FGFRs) [1]. In contrast, till date, it has not been demonstrated that FHFs interact with FGFRs and, due to the lack of mitogenic potential, they have been considered as non-signaling proteins [2].

The FHF subfamily is composed of four proteins: FHF1 (FGF12), FHF2 (FGF13), FHF3 (FGF11) and FHF4 (FGF14) [2]. Each FHF subfamily member is represented by at least two distinct isoforms, generated by an alternative splicing of the first exon leading to proteins that differ at their N-termini [3]. FHFs are most prevalent in the nervous system, however these proteins are also expressed by cardiomyocytes, endothelial cells, osteoclasts and fibroblasts [3–7]. Alterations in FHFs are implicated in a number of diseases, including cancers, neurologic disorders and cardiac arrhythmias [8–14]. Despite the increasing number of reports revealing the consequences of FHFs dysregulation, the mechanisms of their action are currently unknown.

It was demonstrated that when present inside the cells, FHFs may act as cofactors for voltage-gated ion channels [15–20]. FHFs form complexes with intracellular proteins, including IB2, β-tubulin, and NEMO209, however the physiological relevance of these interactions is unknown [4, 21, 22]. Since FHFs lack specific signals for secretion, these proteins have been considered to reside only in the cell interior [1]. However, the release of growth factors, including canonical FGFs (e.g. FGF1 and FGF2) by unconventional secretion or by damaged cells was demonstrated [1, 23–25]. Lin and co-workers have recently found that extracellularly administered FHF2 (FGF13) can stimulate intracellular signaling pathways, raising the possibility that FHF proteins may interact with FGFRs, similarly to canonical FGFs [26].

Here we examined the FHF1-FGFR interplay and its significance in signal transduction and specific cellular responses. We have demonstrated that FHF1 directly interacts with FGFRs, leading to the receptor activation and initiation of intracellular signaling circuits. In contrast to the canonical FGFs, FHF1-induced signaling protects cells from apoptosis, but is unable to trigger cell proliferation and FGFR-dependent glucose uptake. Taken together, our data define FHF1 as typical FGF in terms of FGFR binding and activation, however its biological function seems to differ from that of canonical FGFs.

## Methods

### Antibodies and reagents

The primary antibodies: anti-phospho-FGFR (Tyr653/Tyr654) (p-FGFR) (#06-1433), anti-FGFR1 (FGFR1) (#9740), anti-phospho-p44/42 (Thr202/Tyr204) MAP kinase antibody (p-Erk1/2) (#9101), anti-p44/42 MAP kinase antibody (Erk1/2) (#9102), anti-caspase 3 (caspase 3) (#9662) and anti-poly [ADP-ribose] polymerase (PARP) (#9542) were from Cell Signaling Technology. The primary antibodies anti-FHF1 antibody (#PA5-67182) was from Thermo Fisher Scientific. Anti-tubulin primary antibody (#T6557) were from Sigma-Aldrich. Anti-human IgG (Fc) antibody coupled to HRP (#ab97225) was from Abcam. Horseradish peroxidase-conjugated secondary antibodies were from Jackson ImmunoResearch and chemiluminescent substrate was used for their visualization in the ChemiDoc station (BioRad). Heparin Sepharose resin was from GE Healthcare. Geneticin (G-418) was from BioShop. PD173074 and heparin were from Sigma-Aldrich.

### Cells

Mouse embryo fibroblast cells (NIH3T3) obtained from American Type Culture Collection (ATCC) were cultivated in DMEM (Thermo Fisher Scientific) supplemented with 10% fetal bovine serum (Thermo Fisher Scientific) and antibiotics (100 U/ml penicillin, 100 μg/ml streptomycin). Murine pro B cell line (BaF3) transfected with FGFR1-IIIc (BaF3-R1c) was a kind gift from Dr. David Ornitz from the Department of Developmental Biology, Washington University School of Medicine. The cells were cultured in RPMI-1640 medium (Thermo Fisher Scientific) supplemented with 10% newborn bovine calf serum (Thermo Fisher Scientific), antibiotics (100 U/ml penicillin, 100 μg/ml streptomycin), β-mercaptoethanol (50 nM) and mouse interleukin 3 (IL-3, PeproTech). Human osteosarcoma cell line, U2OS, stably transfected with FGFR1-IIIc (U2OS-R1) was provided by Dr. Ellen M. Haugsten from the Department of Molecular Cell Biology, Institute for Cancer Research (Oslo University Hospital). U2OS-R1 cells were grown in DMEM (Biowest) supplemented with 10% fetal bovine serum (Thermo Fisher Scientific) and antibiotics (100 U/ml penicillin, 100 μg/ml streptomycin and 1 mg/ml geneticin). 3T3-L1 cells were purchased from American Type Culture Collection (ATCC) and cultured in Dulbecco’s modified Eagle’s medium (DMEM, PAN-Biotech GmbH) containing 10% bovine calf serum (Thermo Fisher Scientific) and antibiotics (100 U/ml penicillin, 100 μg/ml streptomycin) at 37 °C in a 5% CO_2_ incubator. To induce adipocyte differentiation, 3T3-L1 preadipocytes were maintained until 90% confluence. Next the medium was exchanged to differentiation Dulbecco’s modified Eagle’s medium (DMEM, PAN-Biotech GmbH) supplemented with 10% fetal bovine serum (Thermo Fisher Scientific), 0.5 mM isobutylmethylxanthine (IBMX), 1 μg/ml insulin and 1 μM dexamethasone (Sigma-Aldrich) for 3 days. Next, adipocytes were maintained in DMEM supplemented with 10% FBS and 1 μg/ml insulin for maturation until day 12.

### Recombinant proteins

Construct encoding short isoform of human FHF1 (FHF1b, called FHF1 in this manuscript) in pDEST17 was a kind gift from Professor F. Nakayama from National Institute of Radiological Sciences, Chiba, Japan. Recombinant, his-tagged FHF1 was expressed in *E. coli* BL21 CodonPlus (DE3) RIL at 25 °C and purified by affinity chromatography using His-Trap column (GE Healthcare) and gel filtration on PD 10 desalting column or HiTrap desalting column (GE Healthcare). Purity and identity of protein samples were confirmed by SDS-PAGE, western blotting and mass spectrometry. FGF1, the extracellular regions of FGFR1, FGFR2, FGFR3, FGFR4 in the form of Fc fusion proteins, and the Fc fragment of human IgG1 were produced as described previously [27–29].

### Spectroscopic studies

Circular dichroism (CD) measurements were performed using a Jasco J-715 spectropolarimeter. Spectra were recorded in a 0.2 mm cuvette at 21 °C, in the wavelength range of 205–260 nm, using a slit width of 2 nm. Protein samples were in phosphate buffer (25 mM H_3_PO_4_, pH 7.3) at the concentration of 53.5 μM.

To determine the thermal stability of FHF1, denaturation curves were acquired following the changes in the ellipticity signal at 227 nm. Measurements were performed at a protein concentration of 0.5 μM in the presence of 0.7 M GdmCl in 25 mM H_3_PO_4_, pH 7.3 in a cuvette of 10 mm path length, using a scan rate of 0.25 °C/min, as described previously [29]. Data were analysed assuming two state denaturation process using PeakFit software (Jandel Scientific Software).

### SPR measurements

The interaction measurements were performed using Biacore 3000 instrument (GE Healthcare) at 25 °C. The extracellular domains of FGF receptors in Fc fusions (in 10 mM sodium acetate, pH 5.0 for FGFR1, pH 5.2 for FGFR2–4) were immobilized on CM5 (at high density) or CM4 (at low density) sensor chip surface (GE Healthcare) at about 9000 RU or 1000 RU, respectively, using an amine coupling protocol. In order to compare the interaction between FHF1 and all of the FGFRs SPR measurements were performed in PBS with 0.05% Tween 20, 0.02% NaN_3_, pH 7.4 on the high density sensor chip. The FHF1 protein (3 μM) was injected at a flow of 30 μl/min. The association and dissociation were monitored for 120 s and 180 s, respectively. The sensor chip surface was regenerated with 10 mM glycine at pH 1.5. The acquired data were analyzed using the BIAevaluation 4.1 software (GE Healthcare).

To determine kinetic constants of the interaction between FHF1 and FGFR1, measurements were performed in PBS with 0.05% Tween 20, 0.2% BSA, 0.02%, NaN_3_, pH 7.4 on the low density sensor chip. A set of dilutions of FHF1 protein at the concentrations ranging from 0.1 μM to 3.2 μM was injected at a flow of 30 μl/min. The association and disassociation were monitored for 120 s and 180 s, respectively. Between injections, 2.5 M NaCl and 10 mM NaOH were applied to regenerate the sensor chip surface. The data were analyzed using the BIAevaluation 4.1 software (GE Healthcare). Equilibrium dissociation constant (K_D_) was calculated from fitted saturation binding curve [30]. Response values from the last 10 s of the association phase were averaged and used to determine the K_D_.

### ELISA

The 96-well Maxisorp F plate was coated with FGF1 or FHF1 (0.05 μM) at 4 °C overnight and additionally blocked with 3% BSA for 2 h at 4 °C. Wells were washed with TBST (50 mM Tris-Cl, 150 mM NaCl, 0.2% Tween-20, pH 7.5) and incubated with FGFR1-Fc, FGFR2-Fc, FGFR3-Fc, FGFR4-Fc and Fc (as a specificity control). Next the plate was extensively washed with TBST and incubated with anti-human IgG (Fc) antibody coupled to HRP at room temperature (RT) for 1 h. Then, the plate was washed five times with TBST and 3,3′,5,5′-tetramethylbenzidine (TMB) (Sigma-Aldrich) was used for spectroscopic detection (absorbance at 450 nm) of specific interactions.

### FGFR1 activation and downstream signaling

Serum-starved NIH3T3 and U2OS-R1 cells were treated with equimolar concentration (6.5 nM) of recombinant FHF1 (160 ng/ml) or FGF1 (100 ng/ml) in the presence of heparin (10 U/ml) (Sigma-Aldrich) and in the presence or absence of FGFR inhibitor 100 nM PD173074 (Sigma-Aldrich) for 15 min. Cells were lysed with SDS sample buffer and lysates were subjected to SDS-PAGE and western blotting.

In experiments with FGF ligand traps serum-starved NIH3T3 were pre-incubated with FGFR1-Fc, FGFR2-Fc, FGFR3-Fc or FGFR4-Fc (10 μg/ml) for 1 h at 37 °C in the presence of 10 U/ml heparin (Sigma-Aldrich). Then, cells were treated with equimolar concentrations (6.5 nM) of recombinant FHF1 or FGF1 for 15 min. The activation of signaling was evaluated by western blotting.

For the analysis of biological activity of FHF1 in cell conditioned media, NIH3T3 cells were starved in a serum-free medium for 24 h. FHF1 and FGF1 in 6.5 nM concentration were added to the medium and incubated with cells for 48 h at 37 °C. Then, conditioned medium was aspirated and added to the new set of serum-starved NIH3T3 cells for 15 min at 37 °C. Activation of cell signaling cascades was used as a sensitive readout of proteins degradation, as described previously [31]. Freshly prepared FHF1 or FGF1 solutions served as positive controls. Cells were lysed with SDS sample buffer and lysates were analyzed with SDS-PAGE and western blotting.

### Cell proliferation

24-h-starved NIH3T3 and BaF3-R1c cells grown on the 96-well plates were treated with increasing, equimolar concentrations (0.065–6.5 nM) of FHF1 or FGF1 in the presence of heparin (10 U/ml). After 48-h incubation at 37 °C number of viable cells was quantified using PrestoBlue Cell Viability Reagent (Thermo Fisher Scientific). The emission of fluorescent reduced form of the dye was measured at 590 nm upon excitation at 560 nm using Infinite M1000 PRO plate reader (Tecan). The proliferative effect was normalized and expressed as a percentage of maximal response observed for FGF1.

### Cell apoptosis and viability

Serum-starved U2OS-R1 cells were treated with serum, 13 nM of recombinant FHF1 or FGF1 in the presence of 10 U/ml heparin. The relative caspase-3/7 activity was measured using ApoLive-Glo Multiplex Assay (Promega) according to the manufacturer’s protocol. The ratio of the caspase-3/7 activity to the cell viability was normalized towards the untreated cells, and denoted as relative caspase-3/7 activity.

NIH3T3 cells cultured in complete medium were treated with 1 μM staurosporine and 13 nM recombinant of FGF1 or FHF1 in the presence of 10 U/ml heparin for 24 h or 48 h. Then the viability was measured using Presto Blue Cell Viability Reagent (Thermo Fisher Scientific) and normalized towards untreated cells.

U2OS-R1 cells were treated with 1 μM staurosporine and 6.5 nM recombinant of FGF1 or FHF1 in the presence of 10 U/ml heparin for 6 h. Cells were lysed with SDS sample buffer and lysates were subjected to SDS-PAGE and western blotting.

### Glucose uptake

Differentiated 3T3-L1 cells seeded on the BioCoat™ Poly-D-Lysine 96-well (Corning) in DMEM without glucose (Thermo Fisher Scientific) were stimulated with two concentrations (0.13 nM and 1.3 nM) of FHF1 or FGF1 in the presence of heparin (10 U/ml) for 16 h. Next, the glucose uptake were determined using the Glucose Uptake-Glo™ Assay (Promega) according to the manufacturer’s protocol. The results were normalized and expressed as a percentage of the signal generated by serum.

## Results

### FHF1 activates FGFR1 and receptor-downstream signaling pathways

**Fig. 1:**
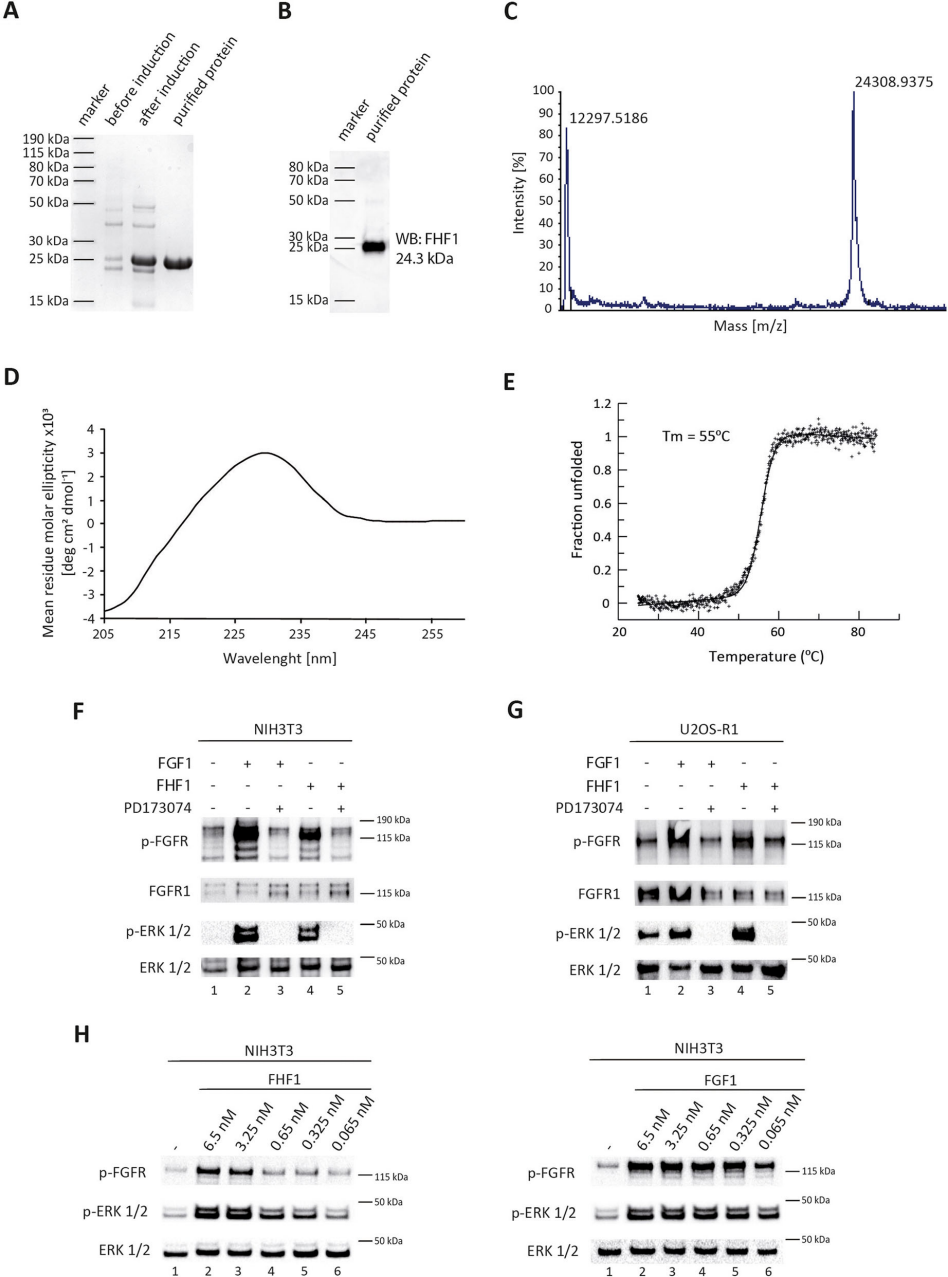
Extracellular FHF1 activates FGFR1. **a** SDS-PAGE, **b** western blotting and **c** mass spectrometry analysis of recombinant FHF1 produced in bacterial expression system (calculated mass 24,302 Da). **d** CD spectrum of FHF1 between 205 and 260 nm. **e** Thermal denaturation curve of FHF1. **f** and **g** Activation of FGFR and downstream signaling by FHF1 and FGF1 in NIH3T3 (**f**) and U2OS-R1 (**g**) cells in the absence and presence of FGFR specific inhibitor (100 nM PD173074) assessed with western blotting. **h** Concentration-dependent activation of FGFR1 and ERKs cascades in NIH3T3 cells by FHF1 and FGF1 verified as in (**f)** and (**g**)

Due to the absence of the secretion signal, FHF1 has been considered as an intracellular protein [1]. Furthermore, FHF1 was unable to stimulate cell division via FGFRs, which supported the hypothesis of FHF1 as an intracellular protein only [32]. Still, a number of proteins, including FGF family members reach the extracellular space via unconventional protein secretion or cell disruption, raising the possibility of the extracellular activity of FHF1. To this end, we adapted a procedure for efficient production and purification of recombinant FHF1 [33]. FHF1 was overproduced in *E. coli* and purified to homogeneity with affinity and size exclusion chromatography (Fig. 1a). The identity of purified protein was confirmed with western blotting (Fig. 1b) and mass spectrometry (Fig. 1c). To study if recombinant FHF1 is in a native state and adopts a β-trefoil structure common for proteins from the FGF family, we subjected purified FHF1 to circular dichroism (CD) measurement. CD spectrum of FHF1 was very similar to that of other FGF proteins, suggesting that obtained FHF1 is properly folded (Fig. 1d). Next, we analyzed the stability of FHF1 using thermal denaturation monitored by CD signal at 227 nm. The assessed denaturation temperature of FHF1 was 55 °C (Fig. 1e) and was over 15 °C higher than the melting point of FGF1 [29].

Next, we tested whether extracellularly administered FHF1 is able to activate FGFRs and downstream signaling. Serum-starved NIH3T3 cells were incubated with FHF1 or FGF1 (positive control) and the activation of FGFRs (pFGFR) and ERK1/2 (pERK1/2) were monitored with western blotting. To assess the FGFR-dependence of ERK1/2 activation, a highly selective FGFR inhibitor, PD173074, was used. Supplementation of cells with either FGF1 or FHF1 led to significant increase in the phosphorylation status of FGFR and ERK1/2 (Fig. 1f, lanes 2 and 4). Importantly, FGF1 and FHF1-induced activation of ERK1/2 was fully dependent on FGFR tyrosine kinase activity, as pretreatment of cells with PD173074 blocked completely the phosphorylation of ERK1/2 (Fig. 1f, lanes 3 and 5). We confirmed these data using U2OS cells stably producing FGFR1 (U2OS-R1) [34]. In agreement with results in NIH3T3 cells, treatment of U2OS-R1 cells with FHF1 led to the effective activation of FGFR1 and ERK1/2 (Fig. 1g). Next, we verified the concentration dependence of FGFR1 and ERK1/2 activation by FHF1. In contrast to FGF1, which reached maximal activation of the receptor at 5 ng/ml (0.325 nM), the highest activity of FHF1 was observed at 80 ng/ml (3.25 nM), which corresponds to 10-fold higher molar concentration (Fig. 1h).

All these data suggest that FHF1 maintains three dimensional structure typical for canonical FGFs and is able to activate FGFR and receptor-downstream signaling cascades in a dose-dependent manner.

### FHF1 directly interacts with FGFRs

**Fig. 2:**
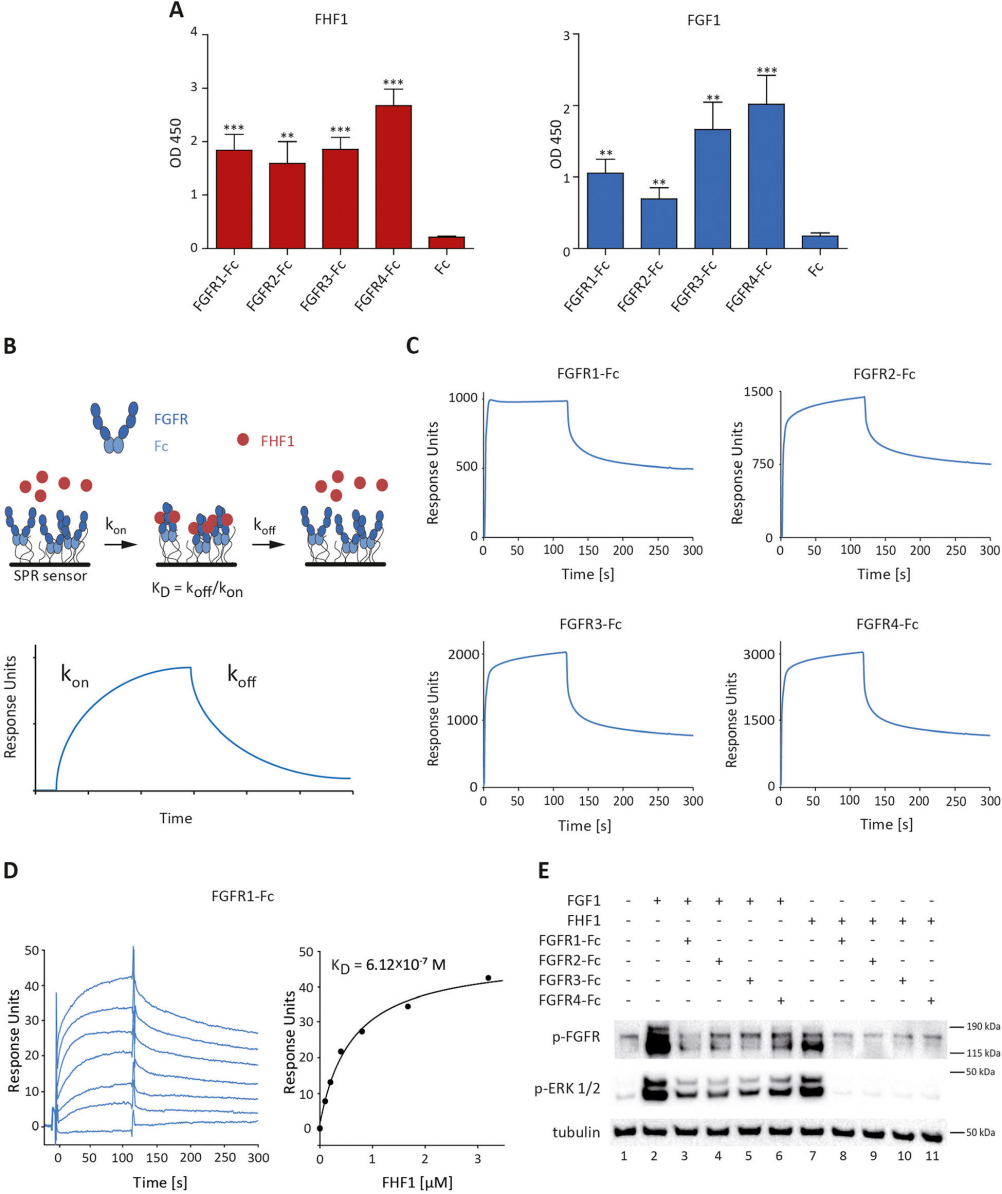
FHF1 directly interacts with FGFRs. **a** ELISA analysis of binding of FHF1 and FGF1 to extracellular domains of FGFRs. Mean values of five independent experiments ± SEM are shown. Student t-test was applied for statistical analysis; ** *p* < 0.01, *** *p* < 0.001. **b** Scheme of SPR experiments using sensor chip with immobilized extracellular part of FGF receptors fused to the Fc fragment. **c** SPR analyses of FHF1 interaction with FGFRs immobilized at high density on CM5 sensor (9000 RU). **d** Kinetics of FHF1-FGFR1 interaction assessed with SPR. The FHF1 protein at the concentrations from 0.1 μM to 3.2 μM was injected on CM4 sensor surface with FGFR1-Fc immobilized at low density (1000 RU). Equilibrium dissociation constant (K_D_) was calculated from saturation binding curve. **e** Soluble extracellular domains of all FGFRs block the activation of FGFR by FHF1 in NIH3T3 cells. Cells were pre-incubated with FGFR1–4 fused to Fc fragment and the ability of FHF1 and FGF1 to trigger cell response was assessed with western blotting

The results of signaling experiments indicated that FHF1 could form a complex with FGFRs. To study FHF1 interactions with FGFRs, we performed enzyme-linked immunosorbent assays (ELISA). Purified FHF1 and FGF1 (positive control) were immobilized on Maxisorp plate and incubated with the extracellular domains of FGFR1-FGFR4 fused with the Fc fragment of IgG (FGFR1-Fc, FGFR2-Fc, FGFR3-Fc and FGFR4-Fc) or with the recombinant Fc fragment (specificity control). Both FHF1 and FGF1 displayed binding to all four FGF receptors (Fig. 2a).

To confirm the direct interaction between FHF1 and four FGFRs we employed surface plasmon resonance (SPR) technique (Fig. 2b). We assumed that recombinant extracellular domains of FGFR1–4 in Fc fusions formed the dimers due to the presence of Fc fragments. Using FGFRs-Fc immobilized at high density on CM5 sensors, we confirmed that FHF1 efficiently interacted with all four FGF receptors (Fig. 2c). Next, we analyzed the kinetics of FHF1-FGFR1 interaction (Fig. 2d). To this end, we injected a set of dilutions of FHF1 protein at the concentrations ranging from 0.1 μM to 3.2 μM on the CM4 sensor with FGFR1-Fc immobilized at low density. The sensograms obtained in SPR experiments revealed a complex nature of FHF1-FGFR1 interaction. The expected interaction model 1:1 (two FHF1 molecules per FGFR1-Fc dimer) did not allow the proper fitting of the data. Therefore to determine K_D_ we employed fitted saturation binding curve derived from equilibrium binding responses plotted against the concentrations of FHF1 (Fig. 2d). K_D_ for FHF1-FGFR1 (6.12*10^− 7^ M) was approximately an order of magnitude lower than that reported for the FGF1-FGFR1 complex [30].

To study whether the activation of FGFR1 and the induction of receptor-dependent signaling cascades is due to the direct interaction of FHF1 with FGFR1, we performed signaling studies in the presence of soluble extracellular domains of FGFRs, acting as ligand traps [28, 35]. The excessive amounts of soluble FGFR1-Fc, FGFR2-Fc, FGFR3-Fc and FGFR4-Fc blocked the FGF1- and FHF1-dependent activation of the cellular pool of FGFRs and ERK1/2 (Fig. 2e). All these data suggest that FHF1 directly interacts with FGFRs, triggering receptor activation.

### FHF1/FGFR-dependent signaling is non-mitogenic and safeguards cells from apoptosis

**Fig. 3:**
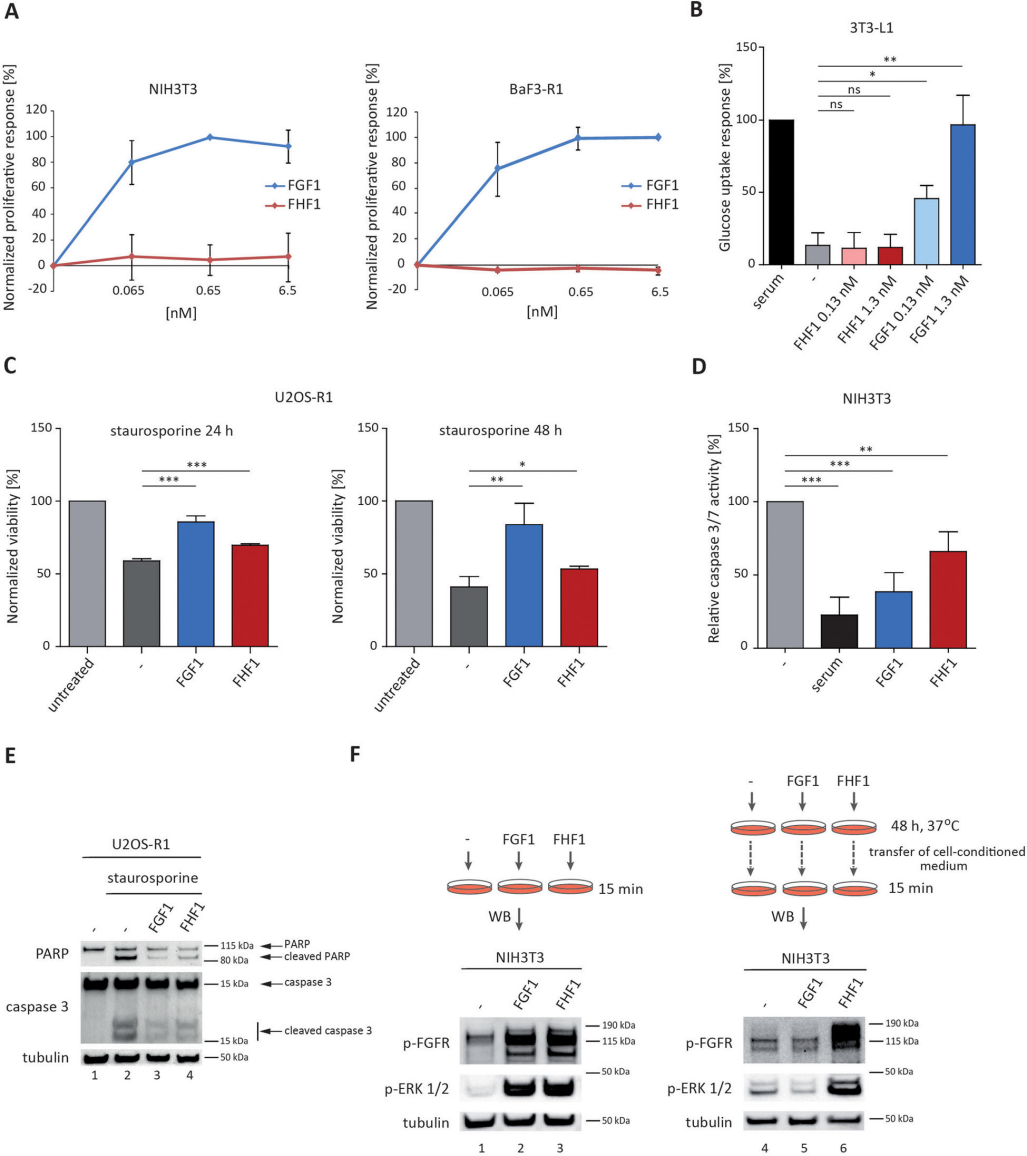
Biological activities of FHF1. **a** Impact of FHF1 and FGF1 on cell proliferation in NIH3T3 and BaF3-R1c cells. The data shown are mean values ± SD of three independent experiments, presented as a percentage of maximal response. **b** Effect of FHF1 and FGF1 on glucose uptake in 3T3-L1 adipocytes. The data shown are mean values ± SD of three independent experiments expressed as a percentage of glucose uptake induced by serum. Student t-test was applied for statistical analysis; * *p* < 0.05, ** *p* < 0.01, n.s. – not significant. **c** Anti-apoptotic properties of FHF1 and FGF1 assessed by measurements of cell viability upon induction of apoptosis with 1 μM staurosporine in U2OS-R1 cells. The data shown are mean values ± SD of three independent experiments expressed as a percentage of viability of untreated cells; * *p* < 0.05, ** *p* < 0.01, *** *p* < 0.001. **d** Ratio of caspase 3/7 activity in serum-starved NIH3T3 cells stimulated with FHF1 and FGF1 to cell viability. The data shown are mean values ±SD of three independent experiments normalized toward untreated cells (relative caspase-3/7 activity). Student t-test was applied for statistical analysis; ** *p* < 0.01, *** *p* < 0.001. **e** PARP and caspase 3 cleavage in U2OS-R1 cells upon induction of apoptosis with 1 μM staurosporine and FHF1 or FGF1 treatment. **f** Long-term stability of FHF1 in cell conditioned media. FHF1 and FGF1 were incubated with NIH3T3 cells for 48 h, and then media containing recombinant proteins were aspirated and tested for their FGFR stimulatory activity with western blotting. Freshly prepared solution of recombinant FHF1 and FGF1 were used as controls

Since FHF1 efficiently activated FGFR-dependent signaling, we addressed the cellular consequences of FHF1-FGFR interplay. It was suggested that the type of cellular response to different FGFs may be dictated by the stability or duration of FGF/FGFR complexes. Short-lived FGF/FGFR signaling units may evoke anti-apoptotic response, while long-lived FGF/FGFR complexes are necessary for induction of cell proliferation [36]. First, we determined the mitogenic potential of FHF1 using NIH3T3 and BaF3-R1c cells. In agreement with the previous studies [31], FHF1 was unable to trigger cell division even at high concentrations (Fig. 3a). In contrast, FGF1, which served as a positive control, stimulated the cell proliferation in a dose-dependent manner.

FGF1 was recently revealed as a novel factor stimulating glucose uptake by adipocytes, playing together with FGF19, FGF21 and FGF23 important role in metabolism [30]. Therefore, we examined the impact of FHF1 on the metabolic activity of 3T3-L1 cells. In agreement with the previous studies [30], we observed that FGF1 stimulated glucose uptake in a dose-dependent manner. In contrast, FHF1 was unable to induce metabolic activity in 3T3-L1 cells at any concentration tested (Fig. 3b).

Next, we studied the impact of FHF1 on the anti-apoptotic activity in model cell lines. U2OS-R1 cells were subjected to staurosporine-induced apoptosis in the presence or absence of FHF1 and FGF1, and cell viability was assessed. Both FGF1 and FHF1 significantly increased cell viability, pointing to the anti-apoptotic activity of these proteins (Fig. 3c). Similarly, FGF1 and FHF1 significantly decreased caspase 3/7 activity in NIH3T3 cells subjected to serum starvation-induced apoptosis (Fig. 3d). We confirmed these findings by monitoring poly [ADP-ribose] polymerase (PARP) and caspase 3 cleavage with western blotting after induction of apoptosis in U2OS-R1 cells with staurosporine (Fig. 3e). In both cases we used antibodies that recognize full length proteins and their large fragment resulting from cleavage.

The lack of mitogenic and metabolic potential of FHF1 raised the possibility that FHF1 quickly loses its biological activity, and thus is unable to trigger cell response requiring long-term stimulation of FGF receptor. Therefore, we analyzed the biological activity of FHF1 and FGF1 after long-term incubation in cell-conditioned media. Recombinant FHF1 and FGF1 were incubated with NIH3T3 for 48 h and cell-conditioned media was tested for their ability to activate FGFR and receptor-dependent signaling with western blotting. As expected, both freshly prepared FGF1 and FHF1 efficiently activated FGFR and ERK1/2 in response to a 15-min stimulation (Fig. 3f, lanes 2 and 3). Strikingly, while FGF1 fully lost its biological activity after 48-h incubation with cells, FHF1 retained its ability to activate FGFR and receptor-dependent signaling cascades (Fig. 3f, lanes 5 and 6). These results are consistent with thermodynamic data indicating increased stability of FHF1, as compared to FGF1. Summarizing, our data demonstrate that FHF1/FGFR interaction is productive and triggers anti-apoptotic response of the cells. FHF1 is not able to induce cell division or metabolic activity, however it is not due to its quick degradation and the lack of stability.

## Discussion

The lack of classical signal sequence directing FHFs to the secretory route and the inability of FHFs to stimulate cell division suggested that these proteins exhibit only intracellular activities [1]. Previous binding studies and structural data indicated that FHFs contain alterations in comparison to canonical FGFs that may hinder their interaction with FGFRs [31, 37, 38]. The first indication of the extracellular function of FHFs was reported by Nakayama and co-workers, who showed that recombinant FHF1, when administered extracellularly, was able to protect the intestine against radiation-induced injury [32]. Recently, Lin et al. suggested that extracellular FHF2 was able to trigger intracellular signaling leading to cell proliferation [26]. However, up to date no evidence for FHF/FGFR complex formation was reported.

Our biochemical and biophysical data clearly demonstrate that FHF1 directly interacts with all four FGFRs, and in consequence, triggers receptor activation and initiates downstream signaling. Our results from SPR binding experiments are in contradiction with the outcome of previous studies and this might be due to the different experimental setup. We used recombinant FGFR1 produced in mammalian cells that is subjected to eukaryotic posttranslational modifications, whereas in previous reports FGFR1 of bacterial origin, devoid of such modifications was applied [37, 38]. The exact nature of FHF1-FGFR interactions requires further studies. Importantly, the biological activities induced by FHF1/FGFR1 and FGF1/FGFR1 differ significantly. While complexes of FGFRs with canonical FGFs generate wide spectrum of cellular responses, including cell division, induction of cell motility, stimulation of glucose uptake and anti-apoptotic activity, the FHF1/FGFR1 complex is only able to evoke cell protective activity. What is the molecular basis of these differences is currently unknown and requires further studies. It was proposed that the stability of FGF/FGFR complexes dictates the cellular outcome of FGFR-dependent signaling [36]. For example, mitogenic response requires stable, long-lasting interaction, whereas short term FGFR activation is enough to trigger other types of responses, such as glucose uptake or anti-apoptotic activity. The lack of mitogenic potential of FHF1 cannot be easily explained by the insufficient protein stability, as FHF1 is thermodynamically more stable than FGF1. Furthermore, 48-h incubation with cells caused the complete loss of FGF1 ability to stimulate FGFRs, while had little effect on FHF1 activity. Thus, it is likely that other factors, like specific co-receptors, signaling kinetics, trafficking of FGFRs, or discrete differences in the strength of signal propagation by individual FGFR-dependent cascades decide about the differences between canonical FGFs and FHF1 in elicited cellular responses. Future studies should further explore this issue.

To fulfill extracellular functions FHF1 has to reach the extracellular space. The data regarding FHF1 secretion are missing. However, the lack of classical secretion signal within FHF1 does not exclude the possibility that FHF1 may be released by cells, either through unconventional secretion or by leaky and damaged cells. The members of canonical FGFs, FGF1 and FGF2 are released by the cells via non-classical secretion that is facilitated by their interaction with cell surface heparans [24, 39, 40]. Furthermore, FGF1 and FGF2 are able to cross endosomal membrane, allowing the extracellular FGFs to translocate to the cytosol and the nucleus [41–43]. Similarly to canonical FGFs, it is likely that FHF1 is capable of crossing cell membranes due to the presence of cell penetrating peptide within its sequence [32, 41]. Besides classical activity of FGFRs at the plasma membrane, these receptors can also signal from diverse intracellular compartments, like Golgi, nucleus or mitochondria [44–47]. Thus, the mechanism of FHF1 secretion, its intracellular trafficking and involvement in the regulation of atypically localized FGFRs awaits further studies.

Summarizing, FHF proteins have long been considered as strictly intracellular proteins unable to bind and stimulate FGFRs. However, our data clearly demonstrate that FHF1, when present outside the cells, directly interacts with FGFRs, contributing to the signal transduction and modulating cell behavior. In this way FHF1 is highly similar to canonical FGFs and therefore, we propose to treat it as a full member of FGF family and use only the name FGF12 instead of FHF1.

## Data Availability

The datasets used in this study are available from the corresponding author on reasonable request.

## Abbreviations

BSA: Bovine serum albumin
CBB: Coomassie brilliant blue
ERK1/2: Extracellular signal-regulated kinases 1/2
FGFR: Fibroblast growth factor receptor
FGF1: Fibroblast growth factor 1
FHF1: Fibroblast growth factor homologous factor 1
MS: Mass spectrometry
TMB: 3,3′,5,5′–tetramethylbenzidine
